# Preliminary Study on Phytochemical Constituents and Biological Activities of Essential Oil from *Myriactis nepalensis* Less.

**DOI:** 10.3390/molecules27144631

**Published:** 2022-07-20

**Authors:** Jikai Fu, Yang Gao, Xiang Xing

**Affiliations:** 1 Sdu-Anu Joint Science College, Shandong University, Weihai 264209, China; jikai_fu@163.com; 2 Marine College, Shandong University, Weihai 264209, China; gygiang@email.sdu.edu.cn

**Keywords:** *Myriactis nepalensis* Less., essential oil, antibacterial, synergistic, cytotoxic, antibiofilm

## Abstract

In response to the need for novel therapeutic strategies to combat the development of microbial resistance, plant essential oils may represent a promising alternative source. This study set out to characterize the chemical composition and assess the antibacterial potential of *Myriactis nepalensis* Less. essential oil (MNEO). Essential oil isolated from *M. nepalensis* by hydrodistillation was analyzed using a GC–MS technique. The antibacterial properties of MNEO alone and combined with antibiotics (chloramphenicol and streptomycin) were tested via the disc diffusion, microbroth dilution, and checkerboard methods. MNEO was represented by oxygenated sesquiterpenes (60.3%) and sesquiterpene hydrocarbons (28.6%), with caryophyllene oxide, spathulenol, humulene epoxide II, β-elemene, neointermedeol, and β-caryophyllene as the main compounds. MNEO exhibited a strong antibacterial effect against Gram-positive bacteria, with MIC and MBC values of 0.039 mg/mL and 0.039–0.156 mg/mL, respectively, and synergistic effects were observed in both combinations with chloramphenicol and streptomycin. Furthermore, the antibiofilm and cytotoxic activities of MNEO were also evaluated. The crystal violet assay was used for quantification of *Staphylococcus aureus* biofilm formation, and an MTT (3-(4,5-dimethylthiazol-2-yl)-2,5-diphenyl tetrazolium bromide) assay was conducted to determine cell viability. The results revealed MNEO could dose-dependently inhibit *Staphylococcus aureus* biofilm formation and possessed potential cytotoxic on both normal and cancer cells (IC_50_ values from 13.13 ± 1.90 to 35.22 ± 8.36 μg/mL). Overall, the results indicate that MNEO may have promising applications in the field of bacterial infections.

## 1. Introduction

As resistant pathogens develop and spread, antibiotic resistance poses a major threat to the effective treatment of a growing number of infections caused by bacteria [1]. The establishment of resistance among bacterial infections is acknowledged as a major global public health problem [2,3]. Therefore, it is imperative to search for novel antimicrobial agents and alternative therapeutic strategies. The study of plant-derived natural products plays an extremely essential part in the development of new therapeutic agents [4]. Antimicrobial compounds that come from plants have been studied closely in the last few years [5].

Under growth conditions, plants produce a large number of secondary metabolites to adapt to biotic and abiotic stresses [6,7]; these metabolites are now well documented to exhibit broad biological functions and play important roles in the plant defense system against pathogenic assaults and environmental factors [8,9,10,11]. Among the plants’ secondary metabolites, essential oils could be good candidates for new antimicrobial agents, as researchers have demonstrated that individual essential oils and their separated components exhibit antibacterial properties against a broad spectrum of microorganisms [12,13]. Essential oils are among the most prevalent naturally occurring antibacterial substances, and they are frequently employed as additives, preservatives, and decontaminants [14]. In addition, combination therapy employing both essential oils and classic antimicrobial agents has proven effective in preventing the development of resistant strains [15]. When such interactions result in synergistic effects, they can also be employed to improve therapy efficiency. Another benefit of such combinations is that lower doses are employed, which reduces side effects and treatment costs [16].

*Myriactis nepalensis* Less. (Compositae) is a perennial herb belonging to the genus *Myriactis* (comprising approximately 10 species) and distributed throughout Southwestern and Southern China, growing at altitudes between 1250 and 3400 m. The plant is 15–100 cm high with a short and procumbent rhizome, erect stem, and simple leaf, with a capitulum type of inflorescence. The flowering and fruiting period is from April to November [17]. To the best of our knowledge, no prior studies have investigated the chemical composition and biological activities of the plant *Myriactis nepalensis* Less. Therefore, the present study aimed to characterize the essential oil composition and evaluate the antibacterial properties of MNEO alone and combined with traditional antibiotics, as well as its antibiofilm and cytotoxic activities.

## 2. Results

### 2.1. Chemical Composition of MNEO

The isolation yield for MNEO was 0.68% (w/w based on air-dried plant material). The phytochemical composition determined by GC-MS is presented in Table 1. Altogether, 78 components, accounting for 96.4% of the total composition of the MNEO, were identified (Table 1). Sesquiterpene compounds dominated the chemical composition of MNEO (88.9%), with oxygenated and hydrocarbon components accounting for 60.3% and 28.6%, respectively. The main components were found to be caryophyllene oxide (10.2%), spathulenol (7.3%), and humulene epoxide II (7.2%), followed by β-elemene (6.1%), neointermedeol (4.5%), and β-caryophyllene (4.1%).

### 2.2. Antibacterial Activity of MNEO

The antibacterial properties of MNEO against ATCC strains were evaluated using the disc agar diffusion method to determine the diameter of inhibition zones (DIZ) and the microtiter broth dilution method to determine the MIC and MBC. Based on the susceptibility testing results (Table 2), the MNEO demonstrated a variable level of inhibitory effect against all bacteria tested. With DIZs ranging from 8.3 ± 0.9 mm (in *Escherichia coli*) to 16.7 ± 1.7 mm (in *Bacillus subtilis*), the disk diffusion assay demonstrated that MNEO exhibited broad-spectrum antimicrobial action. The MNEO showed low activity on the Gram-negative bacteria *Pseudomonas aeruginosa* (MIC = 0.625 mg/mL, MBC = 2.500 mg/mL) and *E. coli* (MIC = 1.250 mg/mL); however, the MBC for *E. coli* was not determined when the concentration of MNEO reached the maximum tested. Conversely, *M. nepalensis* EO presented significant bacteriostatic effects against Gram-positive bacteria (MIC values of 0.039–0.078 mg/mL and MBC values of 0.039–0.156 mg/mL). In addition, based on the MBC/MIC ratios, a bactericidal effect was confirmed for the *M. nepalensis* oil against *B. subtilis*, *S. aureus*, and *P. larvae* (ratios ≤ 4) [18].

### 2.3. Combined Effect of MNEO with Chloramphenicol and Streptomycin

Combination therapy employing both essential oils and classic antibiotics has proven effective in preventing the development of resistant strains and improving therapy efficiency [15,16]. Therefore, the combined effect of MNEO with conventional antibiotics (chloramphenicol and streptomycin) against four pathogens was investigated by using a checkerboard microtiter assay [19]. The fractional inhibitor concentration index (FICI) values were calculated to determine the interaction of MNEO with chloramphenicol and streptomycin, and these are reported in Table 3 and Table 4, respectively. The FICI values in association with chloramphenicol and streptomycin were, respectively, in the range of 0.13–0.56 and 0.12–0.50 against all tested bacterial strains. The mixtures of essential oil and chloramphenicol exhibited either synergism (FICI ≤ 0.5) or an additional synergistic effect (0.5 < FICI ≤ 1). The combined application of MNEO and streptomycin showed synergistic interaction against all the tested bacterial strains (FICIs of 0.12–0.50). The decrease in antibiotic MIC values in the presence of essential oil ranged from 4- to 16-fold. This suggested that the antibiotic activity of chloramphenicol and streptomycin was enhanced when combined with MNEO.

### 2.4. Antibiofilm Activity of MNEO

**Figure 1:**
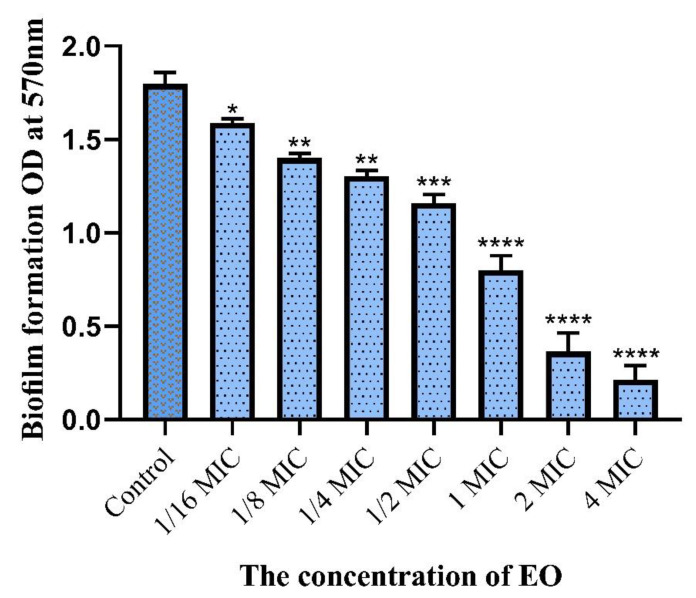
The effect of different concentrations of essential oil from M. nepalensis against biofilm formation of Staphylococcus aureus. Differences were statistically significant in relation to the control for * *p* < 0.05; ** *p* < 0.01; *** *p* < 0.001; **** *p* < 0.0001.

The microtiter-plate technique was conducted to assess *S. aureus* biofilm formation in the presence of MNEO, and a crystal violet (CV) staining assay based on a previously reported methodology was used to quantify biofilm biomass [20]. As shown in Figure 1, *S. aureus* demonstrated a statistically significant reduction in biofilm development when exposed to *M. nepalensis* essential oil. When *M. nepalensis* essential oil at concentrations of 1/16 MIC to 4 MIC was used, 7–88% of *S. aureus* biofilm formation was inhibited.

### 2.5. Cytotoxicity of MNEO

To study the possible cytotoxic activity of MNEO, in vitro metabolic analysis using an MTT colorimetric assay on four human cancer cell lines (colorectal carcinoma HCT-116 cells, hepatocellular carcinoma HepG2 cells, lung adenocarcinoma A-549 cells, and breast cancer MCF7 cells), and one normal cell line (HL-7702 human liver cells) was performed. The cytotoxic activity of MNEO is shown in Table 5 as IC_50_ values. The IC_50_ values obtained from MNEO were 19.53 ± 2.84 μg/mL, 13.13 ± 1.90 μg/mL, 19.19 ± 3.08 μg/mL, and 35.22 ± 8.36 μg/mL for HepG2, MCF-7, A-549, and HCT-116 cells, respectively. For HL-7702, the IC_50_ of MNEO was determined as 19.14 ± 0.63 μg/mL.

## 3. Discussion

As one of the main sources of phytochemical active ingredients, essential oils have been the object of increased interest from researchers, especially regarding their phytochemical profiles and biological activities. Essential oils comprise a range of compounds from diverse classes, including phenolics, terpenoids, aldehydes, ketones, ethers, and epoxides [21,22,23]. These complex mixtures contribute to the broad bioactivity of essential oils. In the present study, the major fraction of MNEO was represented by oxygenated sesquiterpenes (60.3%) and sesquiterpene hydrocarbons (28.6%), with caryophyllene oxide (10.2%), spathulenol (7.3%), humulene epoxide II (7.2%), β-elemene (6.1%), neointermedeol (4.5%), and β-caryophyllene (4.1%) as the main compounds. Among them, β-caryophyllene oxide, β-spathulenol, β-elemene, and β-caryophyllene are the most studied. β-Caryophyllene oxide is frequently found to co-occur with natural bicyclic sesquiterpene β-caryophyllene in many plant essential oils as its metabolite. Several reports have shown that both β-caryophyllene oxide and β-caryophyllene possess significant anti-cancer properties, inhibit the growth and proliferation of numerous cancer cell lines [24,25,26,27,28], and are able to enhance the antiproliferative effect of classical anticancer agents, such as 5-fluorouracil, oxaliplatin, paclitaxel, and doxorubicin [29,30,31,32]. In addition, both of these compounds were found to exhibit anti-inflammatory [33,34], analgesic [26,35], antifungal [36,37], and antibacterial activities [28,38,39].

Spathulenol, a tricyclic sesquiterpenoid with an aromadendrane carbon skeleton, due to its broad spectrum of biological properties, has also been extensively studied in recent years [40], showing antioxidant, anti-inflammatory, antiproliferative, and antimycobacterial activities [41]; insecticidal efficacy [40]; and immunomodulatory response effects [42]. In addition, spathulenol was also demonstrated to be a significantly effective repellent against *Aedes aegypti* and *Anopheles stephensi* [43]. Furthermore, a recent in vivo study showed the acute and persistent antiedematogenic, antihyperalgesic, and anxiolytic activities of spathulenol [44].

Another important compound in *M. nepalensis* essential oil is β-elemene, a noncytotoxic broad-spectrum antitumor drug utilized in Chinese traditional medicine (TCM) for more than 20 years to treat various cancer types, including lung, gastric, cervical, breast, liver, and bladder cancers, etc., including tyrosine kinase inhibitor (TKI)-resistant non-small-cell lung cancer [45,46,47,48,49,50,51,52,53]. Apart from its anticancer activity, β-elemene also possesses antioxidative and anti-inflammatory activities [54,55].

The results obtained from the antibacterial assays demonstrated the promising potential growth-inhibiting effect of MNEO against the tested gram-positive bacteria strains. The tested gram-negative stains were less sensitive to MNEO than tested gram-positive strains, which may be due to the structural differences in their outer membranes. The gram-negative outer membrane is surrounded by an outer layer composed of lipopolysaccharide, which provides an effective barrier of permeability to prevent the diffusion of hydrophobic essential oils [56,57]. The activity of MNEO could be contributed to by the presence of the main components, such as caryophyllene oxide, spathulenol, β-caryophyllene, (Z)-α-santalol, and α-humulene, which exhibit moderate or strong antimicrobial activity [28,39,41,58,59,60,61,62,63,64,65]. However, the possible synergistic effect of all components in the essential oil should also be considered.

Combination therapy using natural and antibacterial agents has been reported as an effective strategy to combat the development of bacterial resistance. Thus, we evaluated the combined effect of MNEO and two conventional antibiotics, namely, chloramphenicol and streptomycin. The most interesting result was obtained for *P. aeruginosa*, for which the MIC value of chloramphenicol was found to decrease from 15.60 to 0.98 μg/mL (FICI = 0.13), and that of streptomycin decreased from 1.95 to 0.12 μg/mL (FICI = 0.12). The most prevalent nosocomial pathogen, *P. aeruginosa*, is intrinsically resistant to numerous drug classes and has the ability to acquire resistance to all available treatment options [66]. Reduced cell permeability, efflux pumps, modifications to the target enzymes, and antibiotic inactivation are some of the primary mechanisms of drug resistance development [67,68]. Moreover, a promising result was also obtained against *S. aureus* (FICI = 0.12)—a 16-fold reduction in streptomycin’s MIC was observed when used with MNEO. The considerable synergistic interaction observed between chloramphenicol and *M. nepalensis* essential oil against the gram-negative *P. aeruginosa* and *E. coli* is also worthy of note. However, the combination of *M. nepalensis* essential oil and chloramphenicol showed only additive interaction when tested against *S. aureus*, as FICI = 0.56 for this combination; it should be considered that the additive manner is similar to a synergistic one, since lower dosages of drugs produce desirable results with additive effects [69].

As far as the microtiter plate biofilm assay results are concerned, interestingly, *M. nepalensis* essential oil revealed a significant effect in inhibiting *S. aureus* biofilm formation at the tested concentrations. Biofilm refers to multicellular surface-attached communities of bacteria embedded in a self-produced extracellular matrix consisting of polysaccharides, protein, and DNA [70]. It is widely acknowledged that bacteria are shielded from antibiotics and the hostile environment of the host by living in biofilms. There is proof that planktonic cells are 1000 times more susceptible to conventional medications than cells in biofilms on biotic or abiotic surfaces. [71,72,73,74,75]. Biofilm is difficult to eliminate once formed, systemic infections are more likely to occur, and the bacteria also become more resistant to treatment with traditional antibiotics [76,77,78]. Among bacteria, staphylococci, especially *S. aureus*, are the major causes of biofilm-associated infections [79,80]. *S. aureus* can colonize and develop biofilms in a variety of host environments [79,80,81]. Bacterial cells in biofilms may evade the host immunological response and tolerate much higher concentrations of antimicrobials compared with planktonic bacteria, making biofilm-related infections particularly difficult to eradicate [82,83,84].

It is worth noting that the biofilm responses to *M. nepalensis* essential oil were demonstrated to be concentration-dependent; even at sub-MICs, the essential oil exhibits inhibitory efficacy on biofilm formation. On the contrary, several studies have demonstrated that biofilm formation can still occur in the presence of sub-inhibitory levels of several conventional antibiotic agents [85,86,87,88,89,90]. This phenomenon was not observed with *M. nepalensis* essential oil, which emphasizes the fact that the essential oil has good therapeutic potential against biofilms.

Given the presence of β-elemene, caryophyllene oxide, β-caryophyllene, and β-spathulenol and their related cytotoxic effects, we also analyzed the metabolic activity of *M. nepalensis* essential oil on four cancer cell lines, as well as its cytotoxicity on a non-cancerous cell line, HL-7702. As expected, the *M. nepalensis* essential oil possessed significant cytotoxic activity on HepG2, MCF-7, and A-549, with IC_50_ values less than 20 μg/mL, and moderate activity on HCT-116. Unfortunately, the essential oil showed a cytotoxic effect on HL-7702 cells, with IC_50_ values of 19.14 ± 0.63 μg/mL. The cytotoxic activity of *M. nepalensis* essential oil could be attributed to the above-mentioned main compounds, as their cytotoxic effects on numerous cell lines have been widely reported [24,25,26,27,28,41,45,46,47,48,49,50,51,52,53,91].

## 4. Materials and Methods

### 4.1. Plant Material

The plant *Myriactis nepalensis* was collected in August 2020 from Badong County in Hubei Province, China. Plant material was identified by Professor Hong Zhao (Shandong University, Weihai, China). Samples (voucher specimen NO.020017) are stored in the herbarium of the Department of Biological Sciences, Shandong University, Weihai, China.

### 4.2. Extraction of Essential Oils

The aerial parts of *M. nepalensis* (300 g) were submitted to hydrodistillation using a Clevenger-type apparatus for 3.5 h to extract the essential oil, then dried with anhydrous Na_2_SO_4_ and kept at 4 °C until use.

### 4.3. Identification of Oil Components

The obtained essential oil was characterized through GC/MS (Agilent Technology 6890/5975C, St. Clara, CA, USA), and the relative percentage amounts of compounds in MNEO were assessed by GC/FID. An HP-5MS capillary column (30 m × 0.25 mm × 0.25 μm, Agilent, St. Clara, CA, USA) was used for the separation. Helium was used as the carrier gas with a flow rate of 1.3 mL/min. A 0.2 μL sample of essential oil was injected with split ratio 50:1, and the temperature was programmed from 60 °C (1 min) to 240 °C (12 min) at a rate of 8 °C/min. MS parameters: EI mode at 70 eV; mass range 50 to 550 amu. The identification of compounds of *M. nepalensis* oil was carried out by comparing their retention indices relative to C_7_-C_30_ n-alkanes and mass spectra with those reported in the literature and by comparing their mass spectra with the NIST and Wiley libraries [92,93,94].

### 4.4. Antibacterial Susceptibility Test

The strains studied are five reference strains of microorganisms from American Type Culture Collection (ATCC) and are representative of gram-positive and -negative strains—namely *Staphylococcus aureus* (ATCC 6538), *Bacillus subtilis* (ATCC 6633), and *Paenibacillus larvae* (ATCC 9545) representative of the gram-positive; and *Escherichia coli* (ATCC 25922) and *Pseudomonas aeruginosa* (ATCC 27853) with characteristics of gram-negative bacteria. The bacterial strains were cultured overnight at 37 °C on Mueller–Hinton (MH) medium before each experimental procedure. Before conducting susceptibility assays, the bacterial suspension was standardized versus 0.5 McFarland turbidimetrically and diluted with MH broth medium to reach the required concentration for each procedure.

A disc agar diffusion assay was applied to antibacterial susceptibility tests [95]. Briefly, filter paper discs were soaked with 20 µL of essential oil (10 mg/mL) or chloramphenicol positive control (1 mg/mL), and placed in triplicate on the plates in which the bacteria were inoculated. The plates were then kept at 37 °C for 24 h in an incubator. The results were determined by measuring the diameter of the zone of growth inhibition (DIZ, in millimeters) and are presented as the means of three measurements.

### 4.5. MIC and MBC Determination by a Microdilution Broth Method

The minimum inhibitory concentrations (MICs) of the essential oil and antibiotics were assessed according to the recommendations of CLSI, using the microdilution broth method in 96-well plates [96]. Stock solutions of the essential oil and antibiotics were prepared with dimethyl sulfoxide (DMSO). In 96-well microtiter plates, 100 µL of essential oil or antibiotic was serially two-fold diluted in Mueller–Hinton broth, and an equal volume of approximately 10^6^ CFU× mL^−1^ bacterial suspension was added in each well. A mixture of culture medium and bacterial suspension acted as a growth control. After 24 h of incubation, to stain the bacteria, 1% 2,3,5-triphenyl tetrazolium chloride aqueous solution was added to each well (20 µL per well). The plates were incubated at 37 °C for 30 min, and the MIC values were visually determined as the minimal concentrations that did not produce a red color.

Samples (100 μL) from the MIC experiment wells with no color change were placed on MH agar plates and incubated for 18–24 h at 37 °C. The minimum bactericidal concentration (MBC) was defined as the lowest concentration at which no bacterial growth was observed.

### 4.6. In Vitro Synergistic Antibacterial Activity by the Checkerboard Method

To evaluate the combined action of MNEO and antibiotics (chloramphenicol and streptomycin), the microbroth checkerboard method was conducted to determine the fractional inhibitory concentration (FIC) index (FICI) [19]. The concentrations tested for each essential oil and antibiotic were selected from 4 × MIC to 1/32 × MIC. A 50 μL volume of essential oil was added at decreasing concentrations into the columns of the 96-well plates, and 50 μL of antibiotic was similarly distributed among the rows. Volumes of 100 μL of bacterial suspensions (10^6^ CFU/mL) were then added to each well. The plates were incubated at 37 °C for 24 h. The FIC values were obtained following the same method for obtaining the MIC values, detailed above. The interaction of the association between the MNEO and antibiotics was evaluated by determining the FICI using the following equation:

(1) $$\mathrm{FICI}=\frac{\mathrm{MIC}\;\mathrm{of}\;\mathrm{EO}\;\mathrm{in}\;\mathrm{combination}}{\mathrm{MIC}\;\mathrm{of}\;\mathrm{EO}\;\mathrm{alone}}+\frac{\mathrm{MIC}\;\mathrm{of}\;\mathrm{antibiotic}\;\mathrm{in}\;\mathrm{combination}}{\mathrm{MIC}\;\mathrm{of}\;\mathrm{antibiotic}\;\mathrm{alone}}$$

The results were considered synergistic if FICI ≤ 0.5 and additive when 0.5 < FICI < 1 [97].

### 4.7. Biofilm Formation Inhibition

The potential of essential oil to inhibit biofilm formation by *S. aureus* (ATCC 6538) was assessed using the microtiter-plate technique [20,98]. The plates were assembled in a process similar to the MIC test. A suspension of approximately 10^6^ CFU/mL *S. aureus* and different concentrations of essential oil (1/4 × MIC to 4 × MIC) were added to each well (100 μL per well) of a 96-well microtiter plate, with 3 parallel wells at each concentration, and the plate was incubated at 37 °C for 24 h. The nontreated bacterial suspension was used as the negative control. After biofilm formation, the supernatant was discarded and the wells were washed thrice with PBS (phosphate-buffered saline) to remove the planktonic and weakly attached cells. The remaining attached biofilms were fixed with 200 μL of methanol per well for 15 min. After discarding the supernatant, each well in the plate was stained with crystal violet (2%) for 10 min and washed with distilled water until the water was colorless. The residual CV, which represented the quantity of biofilm, was dissolved in 95% ethanol (200 μL). Finally, an ELISA microtiter plate reader was utilized to determine the optical density (OD) of each well at 570 nm.

### 4.8. Cytotoxic Activity Evaluation

Four human cancer cell lines (colorectal carcinoma HCT-116 cells, hepatocellular carcinoma HepG2 cells, lung adenocarcinoma A-549 cells, and breast cancer MCF7 cells) and one normal human cell line (HL-7702 human liver cells) were obtained from the Shanghai Institute for Biological Sciences (SIBS, Shanghai, China) and were developed in DMEM and RPMI 1640 (for HL-7702 cells) culture medium supplemented with 2 mM glutamine, 10% fetal bovine serum, 100 units/mL of streptomycin, and 100 units/mL of penicillin, cultured at 37 °C in 5% CO_2_. Cells were detached from the monolayer using 0.25% trypsin for 5 min once cells had grown to near confluence.

The metabolic activity of the cells was detected by an MTT-based assay [99]. Briefly, cells were seeded into 96-well microtiter plates (5 × 10^4^ cells/well) and incubated for 24 h for cell adherens. The essential oil was first dissolved in DMSO (final concentration was 0.1%) and then diluted in culture medium. Subsequently, the cells were treated with essential oil or positive control (doxorubicin) for 48 h. Then, 20 μL of MTT (5 mg/mL) solution was added to all wells for 4 h to form formazan, which was then dissolved in 150 μL of DMSO. Absorbances were read at 570 nm. Cell viability in response to treatment was calculated as a percentage of control cells treated with DMSO at the final concentration 0.1%. The results are expressed as 50% inhibitory concentration of cell growth (IC_50_) values.

### 4.9. Statistical Analysis

All the experiments were carried out in triplicate and the data were presented as the mean ± SD. Data were statistically analyzed using GraphPad Prism 8.0 software (GraphPad Software, Inc., San Diego, CA, USA). One-way analysis of variance (ANOVA) was used to analyze the differences among the treatments, and the level of significance was 0.05.

## 5. Conclusions

The present study investigated the components of *M. nepalensis* essential oil, as well as the potential antibacterial activities of the essential oil, for the first time. The results show that *M. nepalensis* essential oil is rich in caryophyllene oxides (10.2%), spathulenol (7.3%), and humulene epoxide II (7.2%), followed by β-elemene (6.1%), neointermedeol (4.5%), and β-caryophyllene (4.1%). The essential oil of *M. nepalensis* demonstrated antibacterial activities against representative Gram-positive and -negative strains. Furthermore, the ability of *M. nepalensis* essential oil combined with traditional antibiotics to increase the sensitivity of the tested strains to chloramphenicol and streptomycin was investigated. Inhibitory activity was observed against *S. aureus* biofilm formation. Additionally, cytotoxic potential of *M. nepalensis* essential oil was observed on both normal and cancer cells. Considering all the results obtained, *M. nepalensis* essential oil could be a promising alternative for the treatment of various pathogenic bacterial strains; however, further detailed investigations in vitro and in vivo on the biological effects of this essential oil are required to elucidate the mechanism of action and evaluate safety.

## Figures and Tables

**Table 1:** Chemical composition of *M. nepalensis* essential oil.

Peak No.	Compound	RI ^a^	RI ^b^	% Area
1	(*E*)-2-Octenal	1050	1049	1.1
2	Linalool	1098	1095	0.6
3	*cis*-Verbenol	1141	1137	0.1
4	*α*-Terpineol	1190	1186	0.1
5	*cis*-Myrtanol	1252	1250	0.2
6	(*E*)-2-Decenal	1259	1260	0.1
7	Bornyl acetate	1285	1284	0.1
8	Dihydroedulan	1295	1293	0.2
9	Menthyl acetate	1297	1294	0.1
10	Isomenthyl acetate	1306	1304	0.2
11	*δ*-Elemene	1338	1335	0.1
12	7-epi-Silphiperfol-5-ene	1346	1345	0.1
13	*α*-Cubebene	1350	1348	0.1
14	(*E*)-2-Undecenal	1361	1357	0.1
15	Cyclosativene	1369	1369	0.1
16	*α*-Ylangene	1373	1373	0.1
17	*α*-Copaene	1378	1374	0.3
18	*β*-Bourbonene	1387	1387	0.7
19	*β*-Elemene	1393	1389	6.1
20	*cis*-*α*-Bergamotene	1415	1411	0.8
21	*β*-Caryophyllene	1420	1417	4.1
22	*γ*-Elemene	1435	1434	1.0
23	Aromadendrene	1442	1439	0.1
24	*α*-Himachalene	1448	1449	0.1
25	*α*-Humulene	1455	1452	3.2
26	Alloaromadendrene	1460	1458	0.1
27	*γ*-Muurolene	1478	1478	1.5
28	Valencene	1491	1496	1.4
29	Viridiflorene	1493	1496	2.4
30	*α*-Selinene	1499	1498	2.0
31	*α*-Muurolene	1502	1500	0.8
32	*β*-Bisabolene	1508	1505	0.5
33	*γ*-Cadinene	1517	1513	0.7
34	Cubebol	1519	1514	0.1
35	*δ*-Cadinene	1525	1522	1.2
36	*cis*-Sesquisabinene hydrate	1538	1542	0.3
37	Selina-3,7(11)-diene	1543	1545	0.4
38	*α*-Calacorene	1546	1544	0.5
39	Dehydronerolidol	1557	1562	1.2
40	(*E*)-Nerolidol	1563	1561	0.7
41	Mint oxide	1572	1574	0.7
42	Spathulenol	1575	1577	7.3
43	Caryophyllene oxide	1585	1582	10.2
44	Salvial-4(14)-en-1-one	1598	1594	1.8
45	Widdrol	1603	1599	0.7
46	Humulene epoxide II	1609	1608	7.2
47	1-*epi*-Cubenol	1624	1627	1.0
48	epi-*α*-Cadinol	1637	1638	0.7
49	Caryophylladienol II	1641	1639	1.1
50	Isospathulenol	1643	1640	3.2
51	*α*-Muurolol	1645	1644	1.0
52	*α*-Cadinol	1653	1652	0.6
53	Neointermedeol	1660	1658	4.5
54	Zizanol	1672	1677	0.7
55	(*Z*)-*α*-Santalol	1675	1674	3.6
56	Eudesma-4(15),7-dien-1β-ol	1691	1687	2.2
57	Aristol-1(10)-en-9-ol	1708	1704	3.4
58	*β*-Santalol	1719	1715	1.5
59	Vetiselinenol	1726	1730	2.0
60	*γ*-Costol	1749	1745	0.2
61	*α*-Vetivol	1751	1756	0.7
62	*α*-Cyperone	1756	1758	0.3
63	*β*-Acoradienol	1760	1762	0.7
64	*β*-Costol	1768	1765	1.2
65	*α*-Costol	1776	1773	0.3
66	14-hydroxy-*α*-muurolene	1780	1779	0.6
67	(*E*)-Isovalencenol	1788	1793	0.3
68	Neophytadiene	1836	1840	0.7
69	Hexahydrofarnesyl acetone	1843	1847	0.7
70	Rimuene	1901	1896	0.2
71	(5*E*,9*E*)-Farnesyl acetone	1909	1913	0.2
72	Carissone	1927	1926	0.4
73	Verrucarol	1941	1939	0.1
74	Methyl linolelaidate	1976	1980	0.2
75	(*Z*,*E*)-Geranyl linalool	1996	1998	0.4
76	Panaxynone	2022	2018	2.0
77	Thunbergol	2069	2073	0.1
78	Phytol	2108	2114	0.1
	Oxygenated monoterpenes			1.6
	Sesquiterpene hydrocarbons			28.6
	Oxygenated sesquiterpenes			60.3
	Diterpenes hydrocarbons			0.7
	Oxygenated diterpenes			0.6
	Total identified			96.4

^a^ Retention index calculated from n-alkanes (C_7_-C_30_) on an HP-5MS column; ^b^ Retention index data from the literature.

**Table 2:** Diameter of the inhibition zones (DIZ), minimum inhibitory concentrations (MIC) and minimum bactericidal concentration (MBC) of the essential oil of *M. nepalensis* (MNEO).

Strain	DIZ (mm) ± SD		MIC (mg/mL)		MBC (mg/mL)	
	MNEO	Chl	MNEO	Chl	MNEO	Chl
Gram positive						
*B. subtilis* ATCC 6633	14.0 ± 1.2	25.1 ± 1.0	0.039	0.002	0.156	0.004
*S. aureus* ATCC 6538	16.7 ± 1.7	23.6 ± 1.3	0.078	0.002	0.078	0.016
*P. larvae* ATCC 9545	13.4 ± 1.1	28.3 ± 1.7	0.039	0.001	0.039	0.002
Gram negative						
*E. coli* ATCC 25922	8.3 ± 0.9	25.9 ± 0.8	1.250	0.002	>2.500	0.004
*P. aeruginosa* ATCC 27853	10.5 ± 1.3	16.7 ± 0.4	0.625	0.031	2.500	0.250

DIZ, diameter of the inhibition zones (mm) is given as the mean ± SD of triplicate experiments; positive control: Chl, chloramphenicol.

**Table 3:** Fractional inhibitory concentration index (FICI) values of the essential oil of *M. nepalensis* (MNEO) and chloramphenicol combinations.

Microorganism		MICa (μg/mL)	MICc (μg/mL)	FICI
*Bacillus subtilis*	MNEO	39.10	9.76	0.37 (S)
	Chl	3.90	0.48	
*Staphylococcus aureus*	MNEO	78.13	4.88	0.56 (A)
	Chl	3.90	1.95	
*Escherichia coli*	MNEO	1250.00	312.50	0.31 (S)
	Chl	3.90	0.24	
*Pseudomonas aeruginosa*	MNEO	625.00	39.10	0.13 (S)
	Chl	15.60	0.98	

MICa: MIC alone; MICc: MIC combined; Chl: chloramphenicol. S, synergy; A, additivity.

**Table 4:** Fractional inhibitory concentration index (FICI) values of the essential oil of *M. nepalensis* (MNEO) and streptomycin combinations.

Microorganism		MICa (μg/mL)	MICc (μg/mL)	FICI
*Bacillus subtilis*	MNEO	39.00	4.88	0.37 (S)
	SM	0.49	0.12	
*Staphylococcus aureus*	MNEO	78.13	4.88	0.12 (S)
	SM	1.95	0.12	
*Escherichia coli*	MNEO	1250.00	312.50	0.50 (S)
	SM	3.90	0.98	
*Pseudomonas aeruginosa*	MNEO	625.00	39.00	0.12 (S)
	SM	1.95	0.12	

MICa: MIC alone; MICc: MIC combined; SM: streptomycin. S, synergy.

**Table 5:** IC_50_ (μg/mL) ± SD values to the essential oil of *M. nepalensis* and positive control doxorubicin against cell lines.

Cell Line	Essential Oil	Doxorubicin
HepG2	19.53 ± 2.84	0.46 ± 0.02
MCF-7	13.13 ± 1.90	0.70 ± 0.05
HL-7702	19.14 ± 0.63	0.60 ± 0.13
A-549	19.19 ± 3.08	0.48 ± 0.01
HCT-116	35.22 ± 8.36	0.57 ± 0.03

## Data Availability

The data are contained in this article.

## Sample Availability

Samples of the essential oils are available from the authors.
